# Efficient eco-friendly synthesis of carbon nanotubes over graphite nanosheets from yellow corn: a one-step green approach

**DOI:** 10.1038/s41598-024-65893-6

**Published:** 2024-07-16

**Authors:** El-shazly M. Duraia, Mikael Opoku, Gary W. Beall

**Affiliations:** 1 Physics Department, Faculty of Science, Suez Canal University, Ismailia, 41522 Egypt; 2 Department of Chemistry and Biochemistry, Texas State University-San Marcos, 601 University Dr., San Marcos, TX 78666 USA

**Keywords:** Carbon nanotubes, Graphene, Corn, Carbon nanostructures, Pyrolysis, Raman spectroscopy, Jet self-extrusion, Materials science, Condensed-matter physics

## Abstract

The present work reports the synthesis of multi-wall carbon nanotubes (MWCNTs) over graphite nanosheets by an easy and simple approach without using any external catalyst. Simply, yellow corn seeds were thermally annealed in a hydrogen atmosphere at 1050 °C for 3 h without any pretreatments. Notably, the growth of MWCNTs was observed to preferentially occur on the outer surface of the corn shell. This uncomplicated approach not only emphasizes the feasibility of synthesizing carbon nanomaterials using agricultural by-products but also underscores the potential applications of these synthesized materials in various fields. Samples were examined through a comprehensive analysis employing various techniques, including scanning electron microscopy (SEM), Raman spectroscopy, FTIR, X-ray diffraction (XRD), and high-resolution transmission electron microscopy (HRTEM). The findings unveiled the formation of rolled graphene accompanied by the presence of vertical multi-wall carbon nanotubes (MWCNTs) positioned over stacked graphene sheets. This detailed characterization provides insights into the structural features and arrangement of the synthesized materials, paving the way for a deeper understanding of their potential applications. The pyrolysis temperature is a crucial factor in the morphological characteristics of the synthesized carbon nanostructures. While graphene cage-like structures were obtained at 800 °C, small carbon nanotubes were grafted to larger ones and formed three-dimensional hierarchical morphologies when the annealing temperature increased to 900 °C. The growth mechanism of the carbon nanotubes was explained based on the jet self-extrusion of the generated gases through the inherent pores of the corn seeds. The current technique employed in manufacturing MWCNTs shows significant promise as a green synthesis method for producing catalyst-free MWCNTs suitable for industrial applications including sensors and energy storage materials.

## Introduction

Carbon nanostructures such as fullerene, carbon nanotubes (CNTs), and graphene have garnered tremendous interest over the last two decades due to their promising applications in modern technology, including energy storage materials, sensors, nanocomposites, and water purification^1–7^. The fascination stems from their exceptional physical and chemical properties. Notably, the Nobel Prizes awarded to Kroto, Smalley and Curl for fullerene, Iijima for elucidating CNT structure, and Geim for mechanically isolating graphene highlight the significance of these discoveries. However, transitioning these materials from lab scale to industrial scale requires a simple, cost-effective approach. Traditional synthesis methods often involve complex equipment and hazardous chemicals, prompting a shift towards green synthesis methods using sustainable and eco-friendly precursors to reduce environmental impact^8^.

While significant strides have been made in developing green synthesis methods for carbon nanostructures, challenges remain in achieving both high-quality production and environmentally benign processes. Despite efforts to utilize renewable carbon sources like biomass and employ non-metallic catalysts to minimize environmental impact, issues such as maintaining product quality and simplifying synthesis procedures persist. Furthermore, the complexity of optimizing conditions and implementing pretreatment and post-treatment steps for graphene synthesis underscores the need for continued research and innovation in green synthesis techniques. As the field progresses, collaboration among researchers from diverse disciplines, along with advancements in waste management strategies and process optimization, will be essential in realizing the full potential of eco-friendly approaches for industrial-scale production of carbon nanostructures^9,10^.

In the open literature, there are many reports about the synthesis of carbon nanostructures from renewable carbon sources. Biomass (lignocellulosic or non-lignocellulosic) has been used as the carbon source for the synthesis of carbon nanotubes due to environmental issues and the low level of heavy metals and sulphur^9,11^. Although the pyrolysis of biomass in an inert atmosphere can produce carbon nanotubes on a large scale, the quality of obtained carbon nanotubes is a challenge moreover, the procedure involves the use of catalyst nanoparticles, which contaminate the final product and interfere with some applications. The presence of catalyst nanoparticles inside the carbon nanotubes makes post-cleaning treatment a requirement^12^. In general, even though green synthesis methods aim to be eco-friendly, they may still generate by-products or waste materials. Effective waste management strategies need to be developed to minimize any negative environmental impact associated with the synthesis process. Moreover, green synthesis techniques often require optimized conditions, which involve several steps to obtain high-quality graphene. Besides, the pretreatments and post treatments must be applied before and after the graphene synthesis. In the open literature, a great effort is made to produce carbon nanotubes in a green way. For example, Tripathi uses a green plant extract as a green catalyst for the synthesis of the carbon nanotubes^13^. The catalyst is composed of non-metallic materials, ensuring that it exerts no influence on humans and other living organisms throughout the entire process, from manufacturing to disposal and application. However, this method still uses the traditional CVD in the synthesis. Many reports about the green synthesis of carbon nanotubes and graphene using reduced sugar such as glucose or fructose or using ball mill or hydrothermal techniques^14–18^.

Recent advances in catalyst-free synthesis have revolutionized the production of carbon nanotubes (CNTs) by utilizing renewable carbon sources like biomass, thus eliminating the need for metal catalysts that can contaminate the final product^19,20^. Detailed studies of CNT growth mechanisms have provided deeper insights into how specific conditions influence the formation of unique morphologies, such as trumpet shaped and hierarchical CNTs, enhancing their applications in energy storage, sensors, and nanocomposites. These environmentally friendly methods are not only cost-effective but also scalable, addressing practical challenges associated with traditional synthesis techniques. By employing a single-step thermal annealing process in ultra-high purity hydrogen gas, we successfully synthesized high-quality CNTs using yellow corn seeds, demonstrating the potential for large-scale, sustainable production of advanced carbon nanomaterials.

Herein, we introduce a straightforward simple approach for the synthesis of carbon nanotubes over graphitic layers using corn seeds. This single-step method involves the thermal annealing of yellow corn seeds in ultra-high purity hydrogen gas at atmospheric pressure. Trumpet-shaped rolled graphene sheets, carbon nanotubes, and hierarchical carbon nanotubes were successfully grown using yellow corn seeds. The as-prepared samples were characterized by HRTEM, SEM, Raman spectroscopy, FTIR, and XRD. The present discovery is important, and it is expected to have a great impact on the science and technology of carbon nanomaterials.

## Experimental details

The experimental procedure for synthesizing rolled graphene and carbon nanotubes from untreated yellow corn seeds is distinguished by its simplicity. Initially, ten untreated yellow corn seeds (2.94 g) were placed into a ceramic boat and positioned within a three-zone tube furnace. To establish a controlled environment, the tube furnace underwent a 1-h flush with ultra-high purity argon before initiating the heating process. Subsequently, the material was heated at a rate of 15 °C/min until reaching 1050 °C, facilitated by the flow of ultra-high purity hydrogen gas at a rate of 0.2 L/min. The sample was maintained at 1050 °C for 3 h. Following this, the tube furnace was allowed to cool naturally to room temperature, and the resulting black powder was collected for further investigations without undergoing any washing or cleaning processes. The collected materials underwent characterization and analysis, where the morphological features of the as-grown samples were examined using a field emission scanning electron microscope (FE-SEM) Helios Nanolab 400, equipped with EDAX for elemental analysis. Additionally, the crystalline structure was investigated using X-ray powder diffraction, employing a Bruker D8 powder X-ray diffraction unit (with Cu Kα) to collect powder X-ray diffraction data of the samples. Raman spectroscopy was also conducted to provide further insights into the obtained carbon nanostructures, utilizing Horiba Raman equipment with a laser wavelength of 532 nm and a laser power of 1 mW. All measurements were conducted at room temperature. Moreover, morphological structural investigations were carried out using a JEOL 2010F high-resolution field emission transmission electron microscope (TEM), applying an acceleration voltage of 200 kV. The weight loss associated with the samples during thermal treatment was measured using a TA Instrument thermogravimetric analyzer (TGA) model Q50. TGA is a widely utilized analytical technique employed for assessing the thermal stability and composition of materials. In this study, the temperature was incrementally increased at a rate of 10 °C/min, ranging from 25 to 1000 °C, under an argon purge gas flow set at 40 mL/min. This controlled environment ensures precise measurement of weight changes as a function of temperature, allowing for accurate characterization of the thermal behavior and compositional alterations of the samples.

## Results and discussions

**Figure 1:**
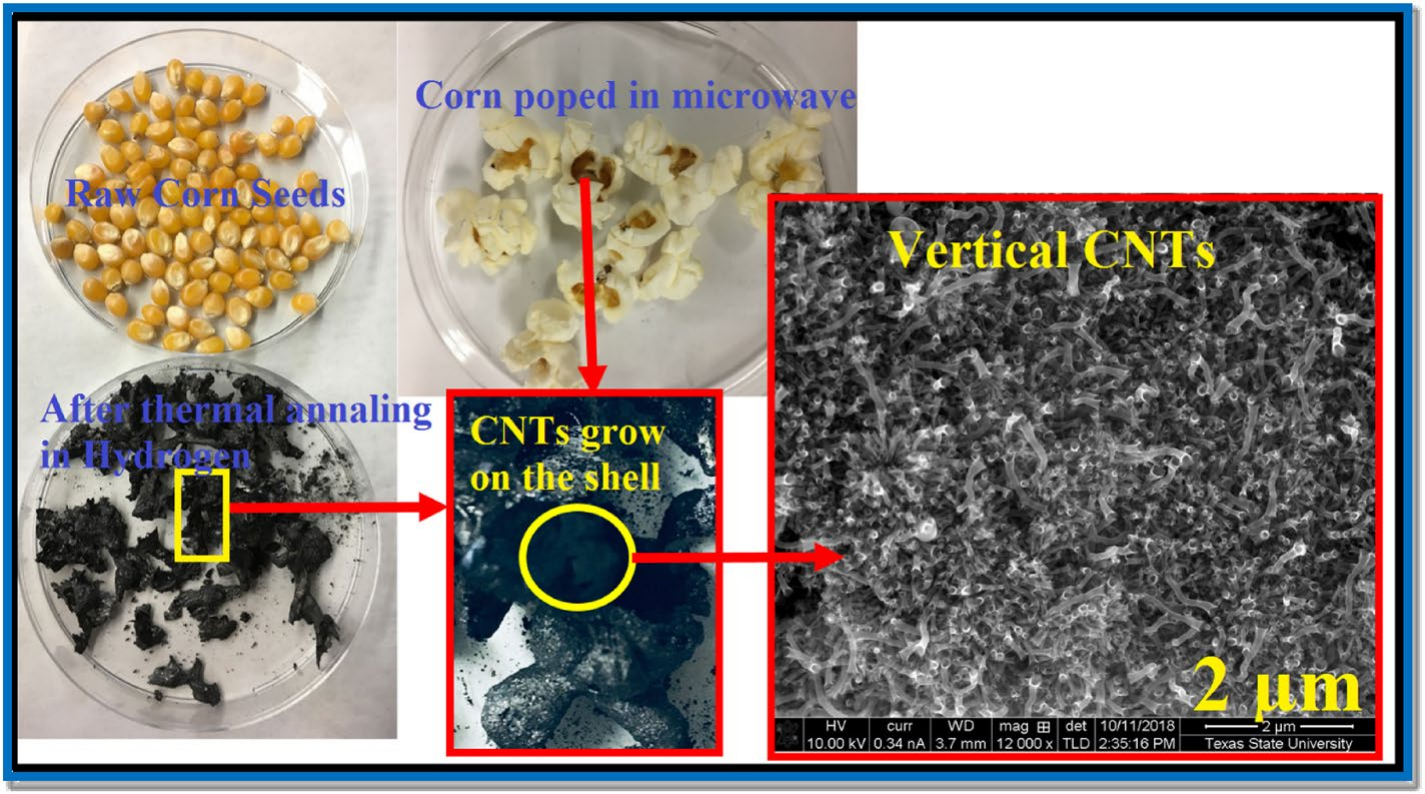
Graphical abstract for the green synthesis of catalyst-free carbon nanotubes by a single step through the pyrolysis of yellow corn seeds inside a hydrogen environment.

Pyrolysis of raw yellow corn seed was performed at three different temperatures (800, 900 and 1050 °C). SEM investigations were performed to investigate the morphological characteristics of the as-grown carbon nanostructures for all samples. Based on the SEM investigations, the morphological characteristics are very sensitive to the pyrolysis temperature. The obtained material was used as it is without further cleaning or post-synthesis purifications. The graphical abstract in Fig. 1 illustrates a one-step, environmentally friendly approach for the synthesis of catalyst-free carbon nanotubes. This process involves the pyrolysis of yellow corn seeds within a hydrogen-rich environment. The schematic provides a visual representation of the key stages, showcasing the transformation of yellow corn seeds into carbon nanotubes in the absence of external catalysts. The green synthesis method is highlighted, emphasizing its simplicity and eco-friendliness. The visual depiction captures the essence of the novel and efficient process, offering a snapshot of the sustainable production of carbon nanotubes from agricultural resources.

**Figure 2:**
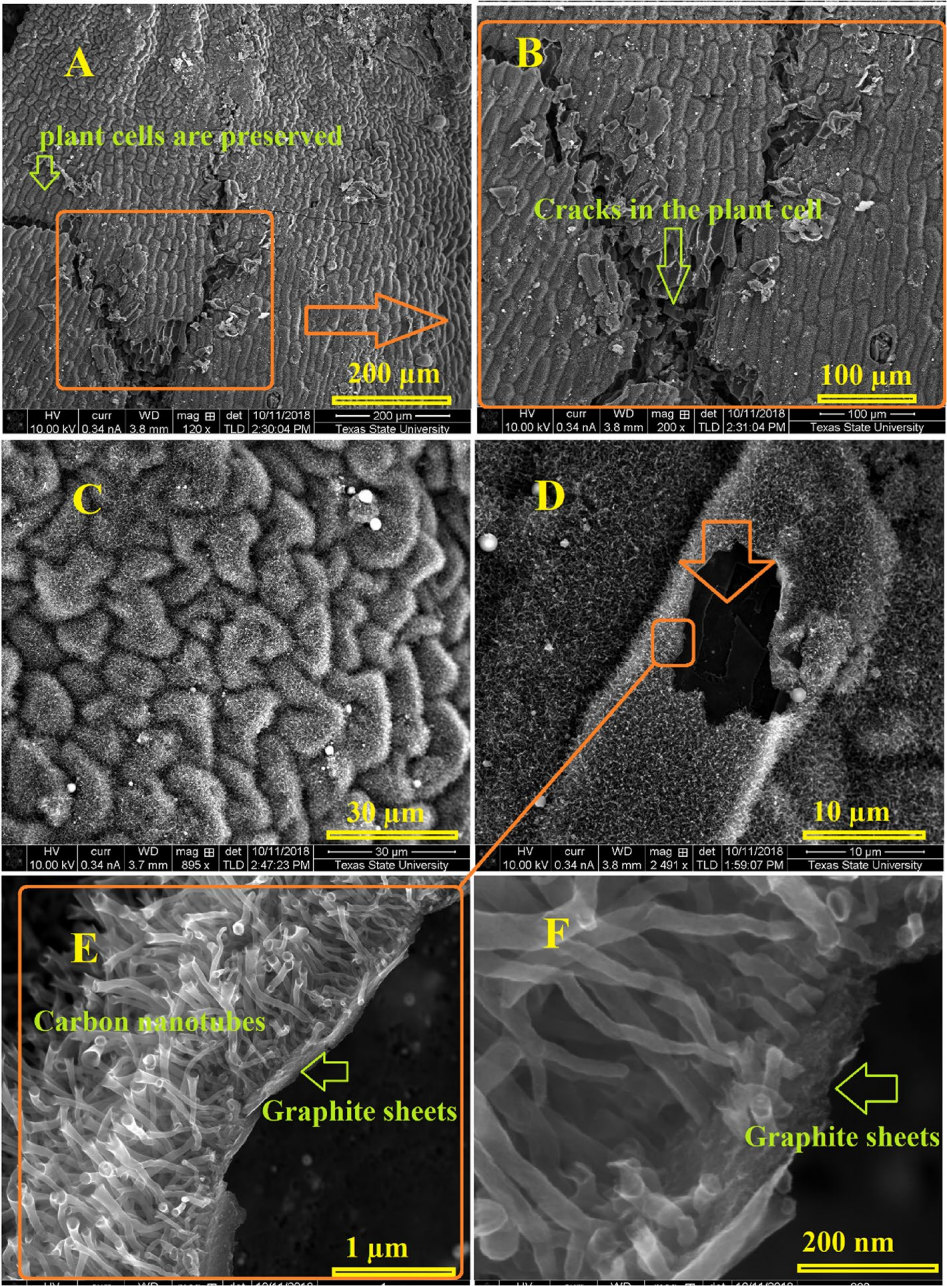
SEM pictures of carbon nanotubes with hierarchical morphology synthesized from corn shell at 1050 °C 3 h in ultra-high purity hydrogen at various magnifications.

**Figure 3:**
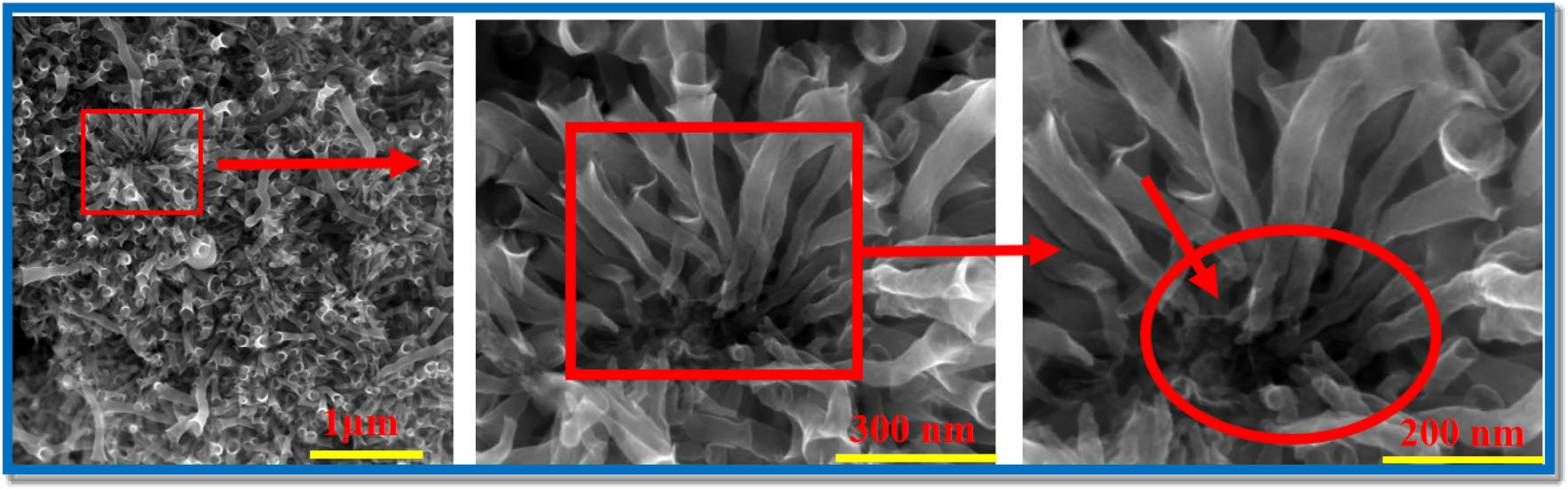
SEM images depicting carbon nanotubes over graphite nanosheets synthesized from corn husks at 1050 °C for 3 h in ultra-high purity hydrogen are presented at various magnifications. The encircled regions highlight the robust attachment of the carbon nanotubes to the graphite sheets.

Figure 2 represents the acquired SEM photographs for the popcorn seeds annealed at 1050 °C for 3 h in in ultra-high purity hydrogen at various magnifications. The low magnification images show the homogeneity growth of the carbon nanostructures which covered the whole surface. Interestingly, a fascinating observation has been made regarding the preferential growth of carbon nanotubes (CNTs) on the outer shell of corn seeds. This distinctive growth pattern is discernible even to the naked eye, evident in the visibly darker black hue of the shell compared to other parts of the popcorn seed. Low-magnification SEM photographs vividly demonstrate the uniformity of CNT growth across a substantial surface area (Fig. 2A). Moreover, the intriguing preservation of traditional plant cell morphology is noteworthy, suggesting that the annealing protocol employed retains the original cellular structure even at elevated temperatures. Given the absence of any external carbon source, it is postulated that all carbon utilized in the growth process originates from the corn itself. Remarkably, the distinctive shape of the corn is maintained even at the high annealing temperature of 1050 °C. The use of ultra-high purity hydrogen gas is deemed crucial in this process, as experiments conducted with Argon or nitrogen resulted in significant carbon material loss during the annealing procedure. Additionally, the observation of rolled graphene nanosheets further contributes to the intriguing findings associated with this innovative synthesis method. Figure 3 illustrates scanning electron microscope (SEM) images capturing rolled graphene nanosheets synthesized from corn shells. The synthesis process occurred at 1050 °C for a duration of 3 h, conducted in an ultra-high purity hydrogen environment. The SEM images were taken at various magnifications, providing a detailed view of the synthesized graphene nanosheets. The morphology of the rolled graphene exhibits a distinctive tubular structure, forming trumpet-like carbon nanotubes. This tubular configuration is characterized by a cylindrical shape with one end expanding into a trumpet-like structure.

**Figure 4:**
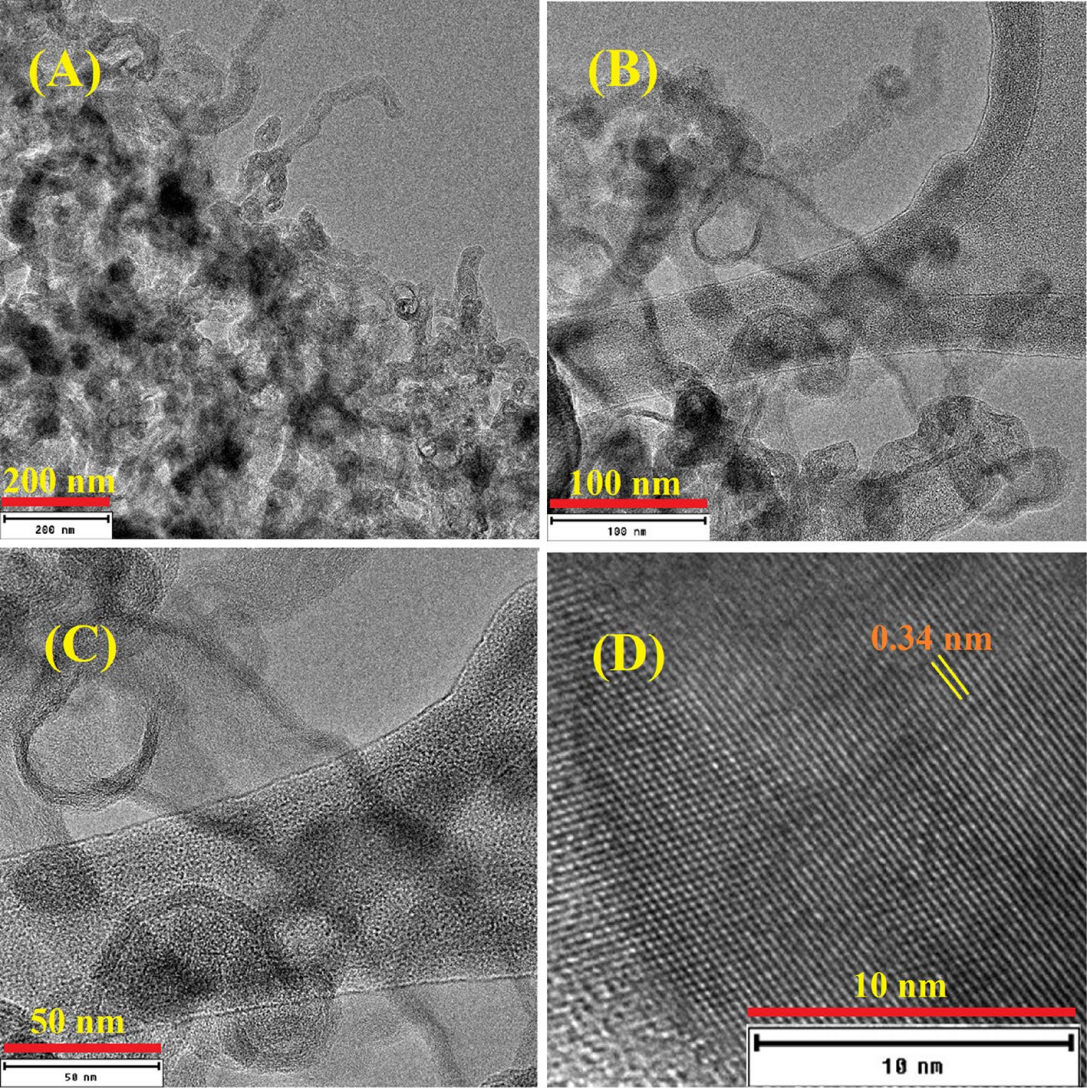
Transmission electron microscope images for the as-grown carbon nanotubes at 1050 °C for 3 h in ultra-high purity hydrogen at different magnifications.

The structural features of the obtained CNT were also studied using transmission electron microscope TEM. Figure 4 represents TEM images for the as-grown carbon nanotubes at 1050 °C for 3 h in ultra-high purity hydrogen at different magnifications*.* Figure 4A depicts the low magnification TEM image showing the formation of CNTs and graphitic nanolayers*.* The TEM demonstrates that the graphene sheets exhibit a short-range order. Moreover, the growth pattern suggests a probable initiation by structural defects situated around the edges of the convoluted paths traversed by venting gas from the substrate. The unique trumpet-shaped carbon nanotube was clearly observed in Fig. 4B,C. In Fig. 4D, the lattice fringe with spacing 0.34 nm can be indexed to the (002) lattice plane for graphite^10,21^.

**Figure 5:**
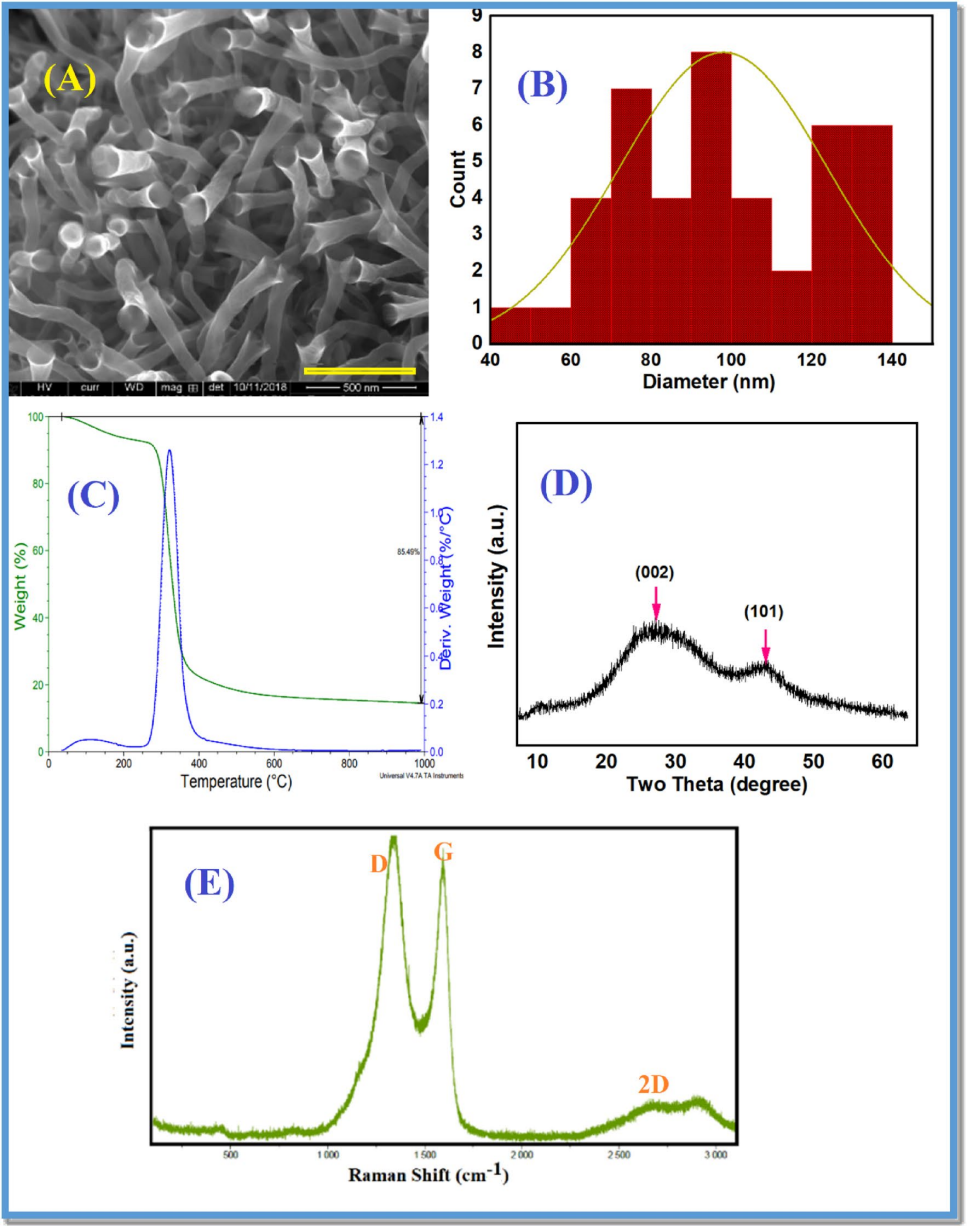
(**A**) Scanning electron microscope image for the as-grown carbon nanotubes at 1050 °C for 3 h in ultra-high purity hydrogen, (**B**) diameter distribution histogram for the carbon nanotubes. (**C**) TGA for the as-grown carbon nanotubes at 1050 °C for 3 h in ultra-high purity hydrogen. (**D**) XRD pattern of the as-grown carbon nanotubes from yellow corn seeds at 1050 °C. (**E**) Raman spectra for the as-grown carbon nanotubes from yellow corn seeds at 1050 °C.

The tube diameter was calculated using ImageJ software (Fig. 5B) based on the SEM images in Fig. 5A. The obtained data revealed that the average diameter is about 100 nm. The weight loss as a function of temperature was estimated using Thermogravimetric Analysis (TGA) over a temperature range of 25–1000 °C, with an argon purge gas flow rate set at 40 mL/min. The TGA curve (Fig. 5C) indicates that the sample experiences significant weight loss around 300 °C, losing approximately 85% of its weight. The remaining 15% of the sample, which is residual carbon, remains thermally stable up to 1000 °C. The investigation into the structural properties of the synthesized nanostructures involved X-ray diffraction (XRD) analysis. The XRD spectrum pattern for the sample annealed at 1050 °C is presented in Fig. 5D. The XRD pattern exhibited the presence of two distinct broad peaks centered at 26 and 43 degrees, corresponding to the (002) and (101) crystallographic planes of graphitic material, respectively^22^.

The determination of surface area was conducted through BET analysis, revealing a remarkable calculated surface area of 710 m^2^/g for the sample subjected to annealing at 1050 °C. This measured value stands out significantly when compared to surface area data reported in the open literature, signifying the notably high porosity and specific surface characteristics of the synthesized material. The obtained surface area underscores the exceptional structural features and potential applications of the annealed sample, showcasing its superiority in comparison to existing literature values^23^. Trumpet-like carbon nanotubes, with their unique morphology and properties, hold great potential for various applications across different fields. Some of the expected applications include energy storage, nanocomposites, and sensors^24^.

Figure 5E represents the Raman spectra for sample annealed at 1050 °C. In general, the D and G bands are major characteristics observed in the spectra of carbon nanostructures, such as graphene and carbon nanotubes. These bands provide worthwhile information about the structural and electronic properties of carbon materials. The G band is associated with the E2g vibrational mode of sp^2^ carbon atoms in a hexagonal lattice. It is a characteristic peak at around 1580 cm^−1^ in the Raman spectra of carbon nanostructures. The G band is often referred to as the “graphitic” band and is related to the in-plane bond-stretching vibrations of *C–C* carbon bonds in a graphene-like structure. The presence and intensity of the G band are indicative of the graphitic nature and the degree of graphitization in the carbon material. Higher intensity and narrower G band suggest a more ordered and crystalline structure. The D band is often referred to as the “disorder” band and is related to the breathing mode of k-point phonons associated with the presence of defects, edges, or other structural disorders in the carbon lattice. The intensity of the D band is proportional to the density of defects and disorder in the material. A higher intensity of the D band suggests a higher degree of disorder and defects^25–29^. In the present case, this ratio was determined to be 1.17, indicating the presence of defects within the material. These defects, highlighted by the elevated D-band intensity relative to the G-band, suggest imperfections in the crystalline structure or the presence of functional groups within the carbon nanomaterials.

The Radial Breathing Mode (RBM) is a characteristic vibration mode that emerges from the cyclical radial expansion and contraction of the individual layers comprising the nanotube structure. The absence or weakening of the RBM peak in Raman spectra reflects a nuanced interplay among several factors intrinsic to the nanotube architecture. This phenomenon results from the intricate dynamics of interlayer interactions, interactions between adjacent tubes, variations in tube diameters across the sample, and the presence of structural irregularities or defects within the nanotube framework. These complex interactions collectively contribute to the alteration or diminishment of the RBM signal, complicating its interpretation and necessitating careful consideration of multiple structural influences when analyzing MWCNT Raman spectra^14,30^.

**Figure 6:**
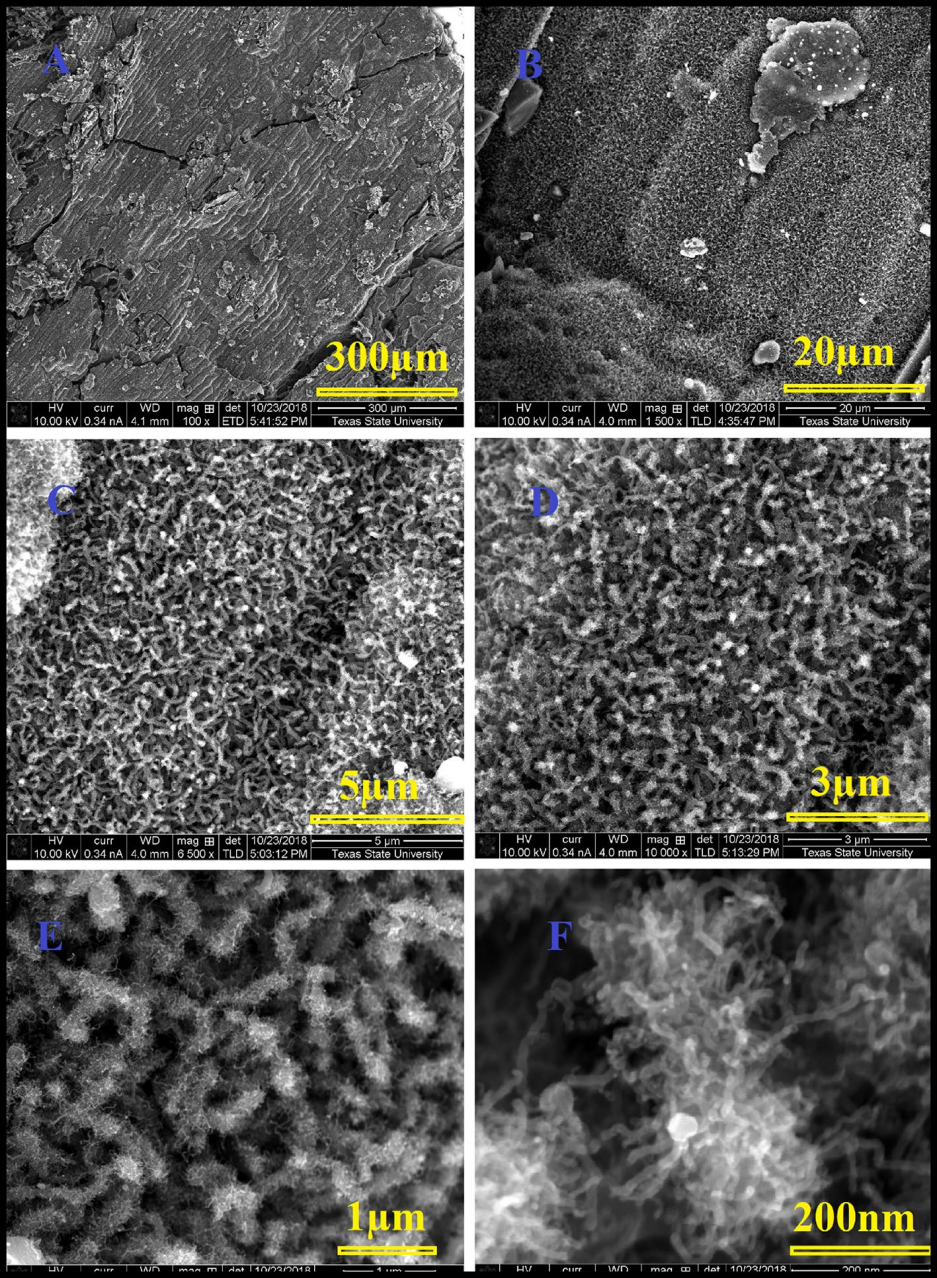
SEM picture of carbon nanotubes with hierarchical morphology synthesized from corn shell at 900 °C 3 h in ultra-high purity hydrogen at different magnifications.

Conducting the exact same experiment at a lower temperature resulted in the formation of entirely distinct carbon nanostructures compared to those obtained under higher temperatures. For example, when the pyrolysis temperature was 900 °C, carbon nanotubes with hierarchical morphology were obtained. In Fig. 6, SEM images of carbon nanotubes with a hierarchical morphology, synthesized from corn shell at 900 °C for 3 h in ultra-high purity hydrogen, are illustrated at various magnifications. The morphological analysis reveals a distinctive structure comprising a central carbon tube with a significantly larger diameter. Notably, smaller carbon nanotubes are observed to grow radially from the surface of this main tube in all directions. This hierarchical arrangement underscores the complexity and unique architecture of the carbon nanotube structures produced during the synthesis process.

**Figure 7:**
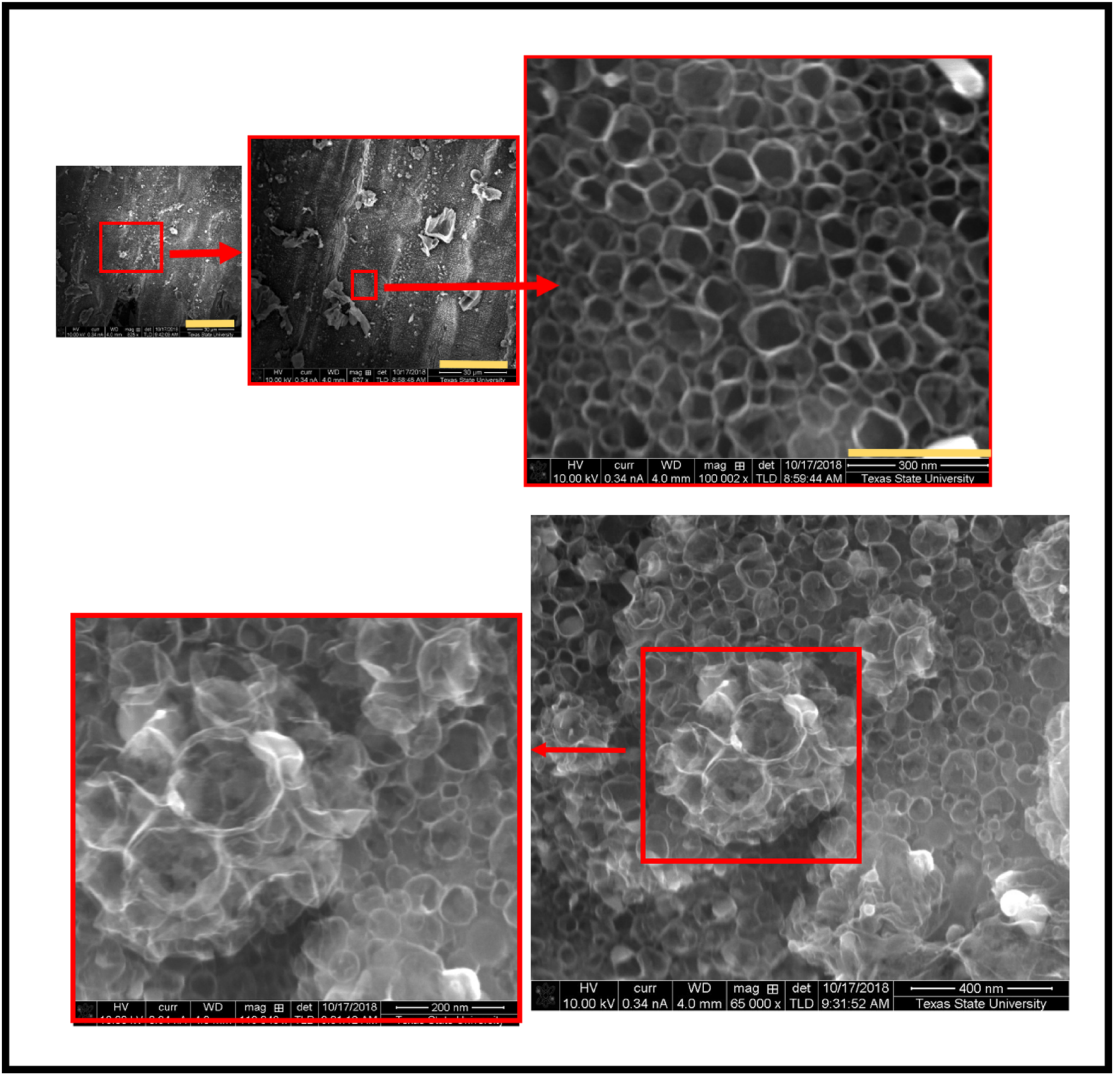
SEM images showing the morphology of carbon nanostructure with cage-like morphology synthesized from corn shell at 800 °C 3 h in ultra-high purity hydrogen in different magnifications.

However, when the pyrolysis temperature decreased to 800 °C, hollow and cage-like carbon nanostructures were obtained. Figure 7 represents the obtained SEM images showing the morphology of carbon nanostructure with cage-like morphology synthesized from corn shell at 800 °C 3 h in ultra-high purity hydrogen in different magnifications. One can easily notice the hexagonal edges of the grown carbon cages. Graphene cage-like structures refer to three-dimensional formations composed of graphene sheets arranged in a manner that creates a hollow or cage-like configuration^31,32^. These structures often exhibit a network of interconnected graphene sheets that encapsulate a central void or cavity. The term “cage-like” implies a spatial arrangement resembling an open framework or lattice, with the graphene sheets forming the boundaries of the cage. The unique geometry of graphene cage-like structures contributes to their distinct physical and chemical properties, making them of interest in various applications, including nanotechnology, materials science, and electronics. The synthesis and manipulation of graphene into such structures offer opportunities for tailoring its characteristics for specific functionalities and applications.

The thermal evolution not only resulted in a shift in the morphology from graphene cages to three-dimensional hierarchical structures but also highlighted the dynamic nature of the annealing process in influencing the formation and interconnection of carbon nanotube structures at different temperature regimes. This observation underscores the potential for temperature modulation as a crucial parameter in tailoring the structural characteristics of carbon nanomaterials for diverse applications.

The choice of corn seeds for this study is attributed to their elevated carbon content, where carbon atoms organize to create amylose (C_6_H_10_O_5_)n and amylopectin. Subjecting this material to thermal treatment within a reducing medium facilitates the elimination of oxygen-containing groups, leading to the development of graphitic nanostructures. The outcomes indicate that the annealing process acts as a catalyst, initiating the formation of a planar hexagonal network characterized by sp^2^ hybridization of carbon atoms. This transformation underscores the potential of corn-derived precursors for yielding graphitic nanostructures, offering insights into the intricate process of carbon rearrangement and hybridization during thermal annealing.

### Proposed growth mechanism of carbon nanotubes

Let's delve into the growth mechanism of trumpet-like carbon nanotubes. The fundamental understanding lies in the crucial role played by catalyst nanoparticles in facilitating the growth of CNTs. Among the most widely recognized catalysts are transition metals, including but not limited to Fe, Ni, Co, and others. These catalysts play a pivotal role in initiating and guiding the carbon nanotube growth process. Their interaction with carbonaceous precursors leads to the nucleation and subsequent elongation of nanotube structures. In the specific case of trumpet-like carbon nanotubes, this mechanism likely involves intricate interplays between the catalyst particles and the carbon feedstock, influencing the unique trumpet-shaped morphology observed in the resultant nanostructures. The exploration of such growth mechanisms not only advances our understanding of nanomaterial synthesis but also holds implications for tailoring carbon nanotube structures for diverse applications^33–36^.

Nevertheless, it has been demonstrated that any nanoparticle can serve as an active site for the growth of carbon nanotubes. For instance, well-aligned horizontally single-wall carbon nanotubes were successfully grown using different metals (Fe, Co, Ni, Cu, Pt, Pd, Mn, Mo, Cr, Sn, Au, Mg, and Al)^37,38^. Fundamentally, the growth of carbon nanotubes in the absence of an external catalyst and carbon source gas is highly dependent on the inherent minerals and volatile matter in biomass. Moreover, the presence of some elements such as silicon or calcium may facilitate the formation of carbon nanospheres and subsequent carbon nanotubes.

**Figure 8:**
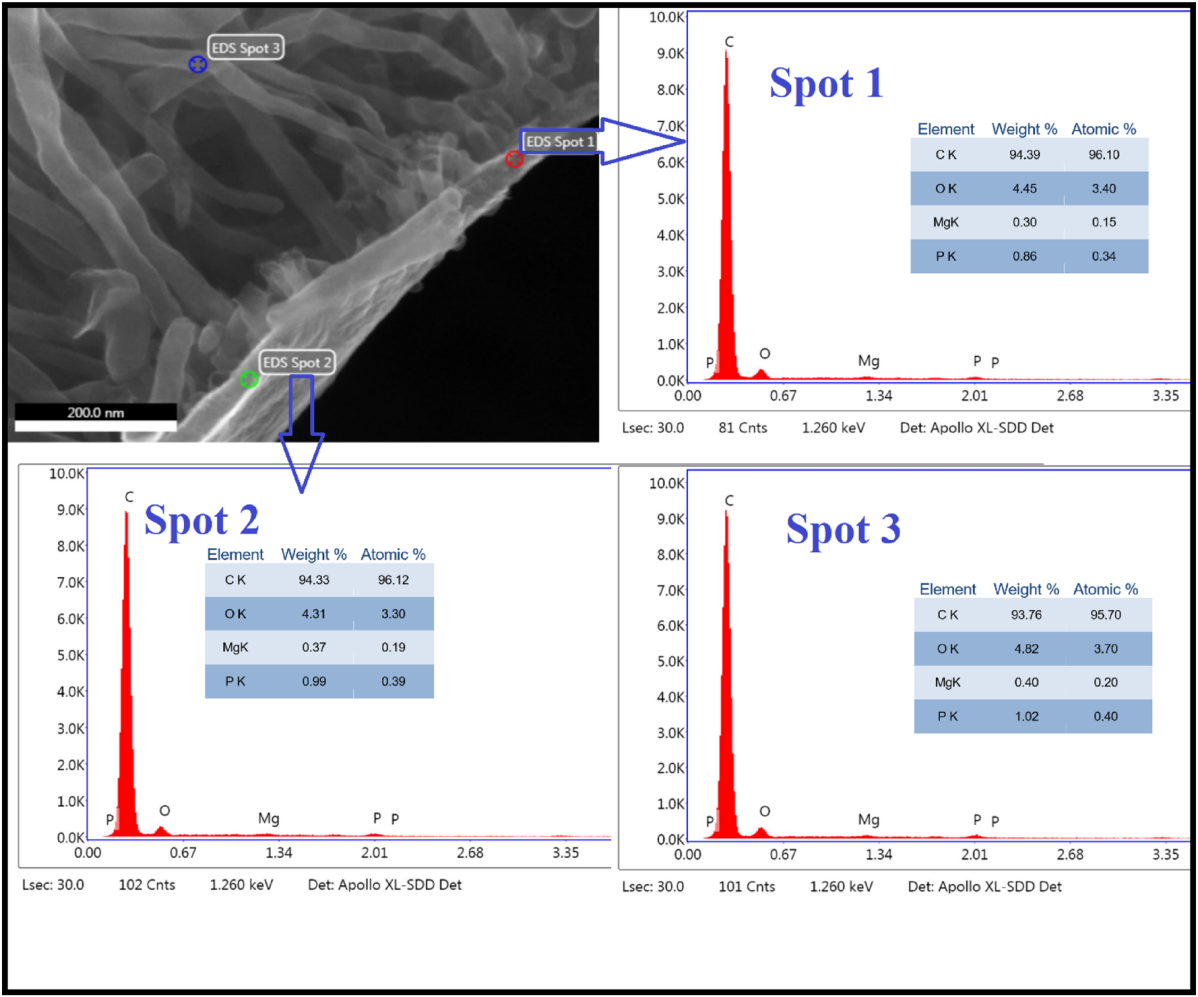
Energy dispersive X-ray spectroscopy for the carbon nanotubes synthesized from yellow corn showing the elemental analysis at three different spots.

According to the EDAX findings in Fig. 8, the analysis reveals the presence of only three elements: carbon (C), oxygen (O), and phosphorus (P). Remarkably, no other elements were identified either at the tip or the base of the carbon nanotube. This observation leads to the implication that the typical growth mechanisms may not be applicable in this instance, given that no external catalyst or additional carbon source was introduced. The absence of diverse elemental signatures, especially in critical regions like the top and bottom of the nanotube, challenges conventional expectations and suggests a unique growth process that differs from established models relying on external catalysts or supplementary carbon feedstock. This novel insight opens avenues for further exploration into the unconventional growth dynamics of these carbon nanotubes.

**Figure 9:**
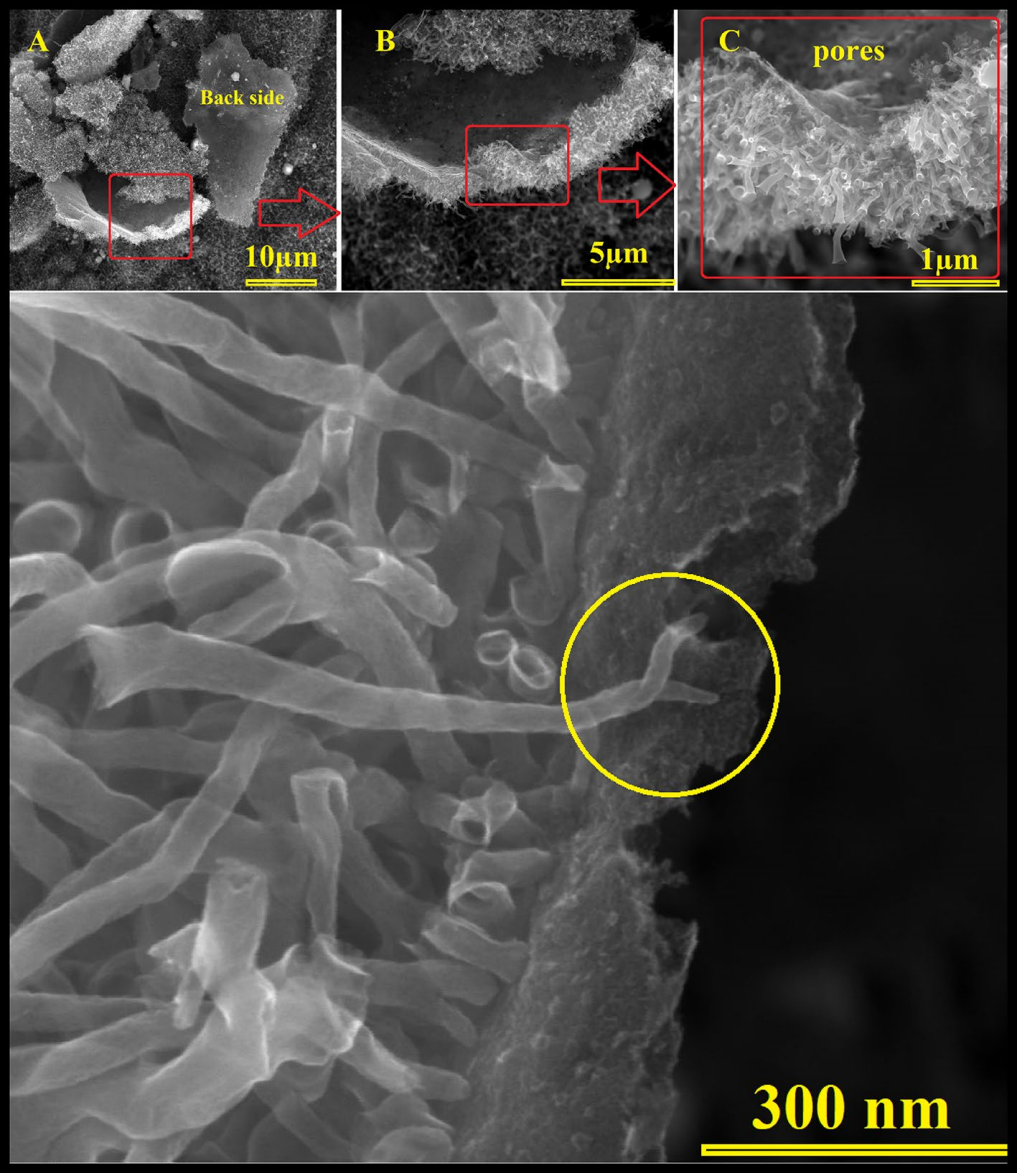
SEM image of the trumpet-like carbon nanotubes showing the roots of the nanotubes. The circle highlights the twisted tubes which support the self-extrusion of the gases from the bulk through the tube, which leads to the open tube end and forms the trumpet-like morphology. The encircled region highlights the robust attachment of the twisted carbon nanotubes to the graphite sheets.

As is seen in Fig. 9 the grown carbon nanotubes are well attached to the graphitic layers. Generally, providing strong attachment of carbon nanotubes (CNTs) to the graphitic layers is very important and crucial for optimizing their performance for many applications. This connection will enhance structural stability, facilitate efficient charge transport, and improve electron mobility. This strong attachment is especially important in electronics for its impact on electrical conductivity. The synergistic effect between CNTs and graphite is favorable and desired in many applications including energy storage, conversion, and sensors due to the fast electron transfer.

The attachment of carbon nanotubes to the graphitic layers may suppose that these carbon nanotubes originate from the graphitic layers. The formation of these carbon nanostructures involves two stages, decomposition, and reconstruction stages. In the absence of ultra-high purity hydrogen gas, decomposition is more than reconstruction.

However, using hydrogen gas helps to preserve most of the carbon structures at the early stages. Hydrogen gas played the most important role in this process which terminates the dangling bond and helps to etch the excess amorphous carbon that may form during the decomposition and growth processes. Moreover, from the experimental observation, it has been noticed that the evaporation of a lot of gases during the decomposition process at around 500 °C was also confirmed by using TGA. We suggest these gases may be the reason for the growth of such morphology or what we called “volcano theory”. Indeed, additional theoretical and experimental work is needed to confirm this theory. Hydrogen atoms are extremely small and can diffuse everywhere, especially at the high temperature used in this experiment. Additionally, the decomposition process involves the liberation of large quantities of gases. These gases that formed between the graphitic layers need to come out from the weakest point in the same manner as a volcano. The other carbon atoms will continue to grow at the edge of these volcano openings. This growth mechanism may account for the trumpet shape of the formed rolled graphene sheets. Almost all carbon nanotubes have exhibited this unique tip opening morphology. This tip opening morphology suggests that gases come out at a high rate and pressure. A similar mechanism called the self-extrusion mechanism was postulated by Omoriyekomwan to explain the synthesis of carbon nanotubes via microwave heating of biomass^9^. In which the volatile matter created during the pyrolysis procedure is pushed or squeezed out through small opening pores (self-extruded) from the bulk core to the surface. The volatile matter then experiences re-solidification due to condensation and because of a temperature reduction due to Joule- Thompson cooling on the particle surface.

To get better understanding about the growth mechanism of such carbon nanostructures, SEM was used to investigate the backside of the grown carbon structures as shown in Fig. 9 as indicated in the figure the carbon nanotubes are well attached to the graphitic layers and pores were observed on the backside which supports the volcano theory.

**Figure 10:**
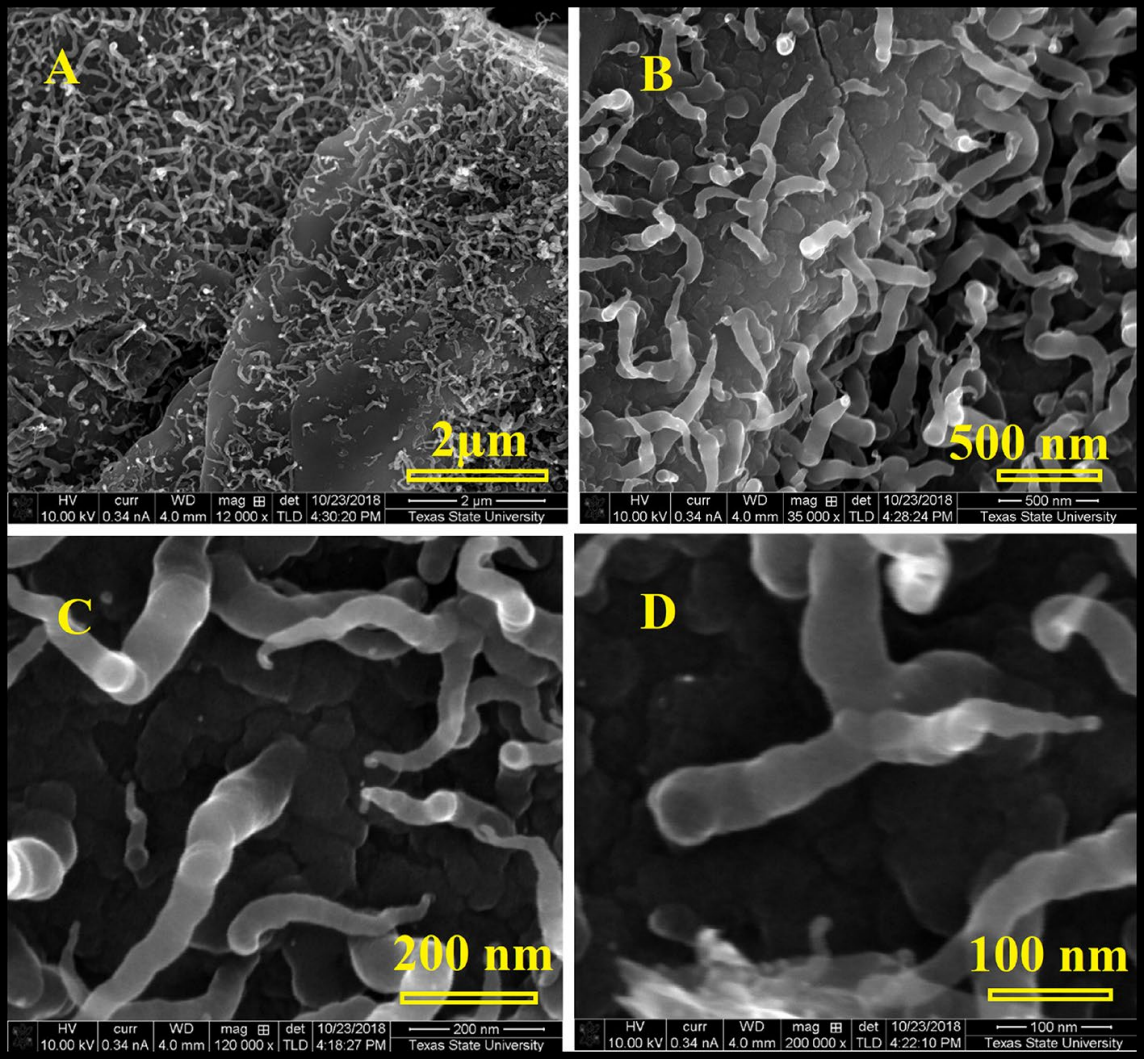
SEM images showing the early stages of the grown carbon nanotubes at different magnifications, (**A**–**D**) the nanotubes are not straight and have a worm-like morphology. Moreover, the diameter of the nanotubes gets narrow at the end. Note the point of attachment of carbon nanotubes to the base, there is a strong connection between the carbon nanotubes and the graphite layers. The annealing temperature was 1050 °C.

**Figure 11:**
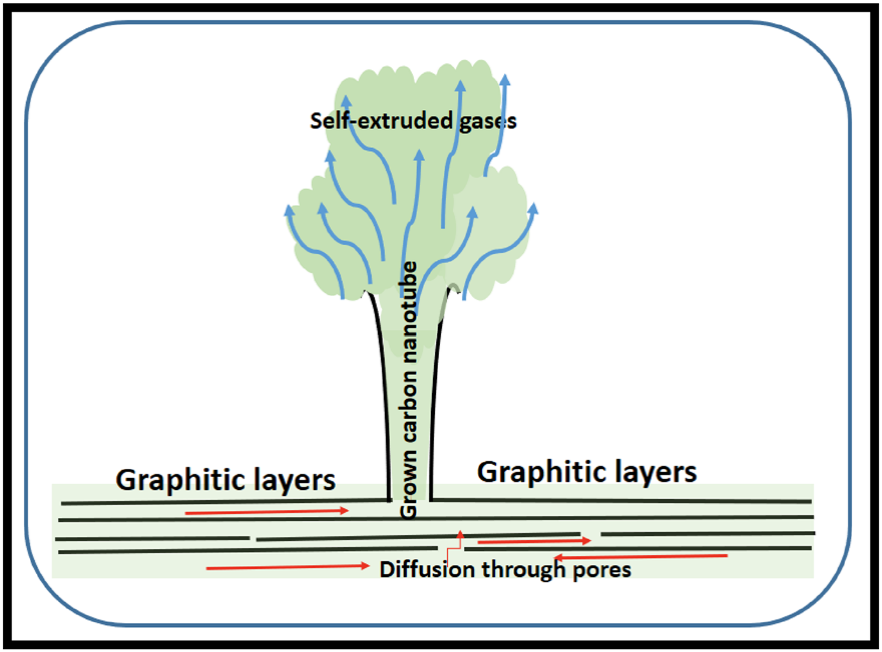
Schematic diagram showing the proposed growth mechanism of the trumpet-like carbon nanotubes by the self-extrusion of the volatile gases through the pores.

Figure 10 represents the SEM micrographs of the obtained nanotubes at the early stages. The carbon nanotubes have worm-like morphology and are not straight with an unusual, closed end. The diameter of the carbon nanotubes is not uniform, but it gets narrower at the tube end. This consequence supports the proposed mechanism. We believe that nanotubes grow only at places where there are pores inside the graphitic layers. The existence of inorganic elements like P will facilitate the formation of carbon nanospheres. Nevertheless, the obtained trumpet-like morphology, as well as the severely twisted tubes, suggested that the generated gas extruded so strongly that it opened the tube end which agrees with Omoriyekomwan’s proposed mechanism. A schematic diagram showing the proposed growth mechanism of the trumpet-like carbon nanotubes by the self-extrusion of the volatile gases through the pores is presented in Fig. 11. The provided schematic diagram illustrates the envisioned growth mechanism of trumpet-like carbon nanotubes, showcasing the self-extrusion phenomenon of volatile gases through the pores. This proposed growth mechanism delineates the process by which the tubular structures emerge, resembling trumpets, because of the self-extrusion of volatile gases through specific pores. The diagram serves to elucidate the intricate steps involved in the formation of these unique carbon nanotubes, providing a visual representation of the self-extrusion process occurring within the nanoscale framework.

Overall, the carbon nanostructures investigated in this study exhibit versatility for a wide range of applications, including electronics, sensors, energy storage, and composite materials. With their unique properties, such as high conductivity and mechanical strength, these nanostructures hold promise for enhancing the performance of various technologies. They could be utilized in electronics for transistors and flexible displays, in sensor technologies for gas and biosensors, and in energy storage devices like supercapacitors and batteries. Additionally, they offer potential as reinforcing agents in composite materials, contributing to improved mechanical and electrical properties. As research progresses, these carbon nanostructures are anticipated to play an increasingly important role in advancing multiple industries and technologies.

## Conclusions

This study has successfully synthesized catalyst-free carbon nanotubes (CNTs) over graphite nanosheets in a single-step process using yellow corn seeds, without requiring pre- or post-treatments. Characterization of the synthesized samples was performed using XRD, SEM, TEM, TGA, and Raman spectroscopy, revealing that the carbon nanomaterials exhibited sensitivity to annealing temperature, forming vertical CNTs with a distinctive trumpet-like shape at 1050 °C. Future research will focus on optimizing synthesis parameters to enhance CNT quality and yield, scaling up the process while maintaining consistency, and exploring functionalization for specific applications. The main challenges anticipated include ensuring scalability without compromising quality and managing variability in raw biomass material, which will be addressed through systematic scale-up studies and standardized pre-processing protocols. Key novel contributions include the catalyst-free synthesis, single-step process, environmentally friendly approach, unique morphology, and versatility for various applications. This work advances the understanding of carbon nanotube synthesis by elucidating the role of annealing temperature and atmosphere in catalyst-free CNT formation, offering an efficient and sustainable method for industrial production with significant potential for applications in nanocomposites, energy storage materials, and sensors.

## Data Availability

We declare that we will make all raw data available. Please contact the corresponding author, El-shazly M. Duraia, at “shazly_duraia@yahoo.com” if any data is requested.
